# Knockdown of miR393 Promotes the Growth and Biomass Production in Poplar

**DOI:** 10.3389/fpls.2021.714907

**Published:** 2021-07-14

**Authors:** Liwei Chu, Xuejiao He, Wenbo Shu, Lijuan Wang, Fang Tang

**Affiliations:** ^1^State Key Laboratory of Tree Genetics and Breeding, Key Laboratory of Tree Breeding and Cultivation of the National Forestry and Grassland Administration, Research Institute of Forestry, Chinese Academy of Forestry, Beijing, China; ^2^Co-innovation Center for Sustainable Forestry in Southern China, Nanjing Forestry University, Nanjing, China; ^3^Key Laboratory of Horticultural Plant Biology of Ministry of Education, College of Horticulture and Forestry Sciences, Huazhong Agricultural University, Wuhan, China

**Keywords:** miR393, STTM, plant growth, auxin signaling pathway, RNA-seq, poplar

## Abstract

Short tandem target mimic (STTM), which is composed of two short sequences mimicking small RNA target sites, separated by a linker of optimal size, can block the functions of all members in a miRNA family. microRNA393 (miR393), which is one of the conserved miRNA families in plants, can regulate plant root growth, leaf development, plant architecture, and stress resistance. In order to verify the role of miR393 in the secondary growth of trees, we created its STTM transgenic poplar lines (STTM393). The expression of miR393 in STTM393 lines was reduced by over 10 times compared with the control plants. STTM393 lines showed promoted growth with about 20% higher, 15% thicker, and 2–4 more internodes than the control plants after 3 months of growth. The cross-section of the stems showed that STTM393 lines had wider phloem, xylem, and more cambium cell layers than control plants, and the lignin content in STTM393 lines was also higher as revealed by staining and chemical determination. Based on the transcriptome analysis, the genes related to the auxin signaling pathway, cell cyclin, cell expansion, and lignin synthesis had higher expression in STTM393 lines than that in control plants. The higher expression levels of *FBL* family members suggested that the auxin signaling pathway was strengthened in STTM393 lines to promote plant growth. Therefore, the knockdown of miR393 using the STTM approach provides a way to improve poplar growth and biomass production.

## Introduction

Poplar is one of the important carbon-neutral biomass for the production of timber products, paper pulping, chemicals, and biofuels (Gui et al., 2020). At present, there is a large demand for wood, and cultivating fast-growing trees like poplar is a promising way to solve this problem. To further improve poplar growth and biomass production, substantial efforts have been made to identify the functions of genes and small RNAs that regulate poplar growth (Lucas et al., 2013; Sundell et al., 2017; Busov, 2018). MicroRNAs (miRNAs) can negatively regulate the transcription and post-transcription of their specific target genes by binding their near-perfect complementary sites through its 20–24 nucleotides, which can lead to the target mRNA degradation and/or translational repression (Meyers et al., 2008; Voinnet, 2009).

In previous studies, in order to understand miRNA functions, the major approaches were used by generating the transgenic lines in which the genes that encode miRNAs or the target gene of miRNAs were overexpressed (Zhu et al., 2009; Zhang et al., 2010; Curaba et al., 2013). However, these approaches were insufficient to fully understand miRNA functions. First, one miRNA often targeted multiple genes for regulation, and modifying one target gene could not possibly reveal the functions of other target genes (Bartel, 2009). Second, overexpressing the miRNAs could only result in a decrease in the expression level of miRNA target genes and an inability to obtain the information of gain of functions of the target genes. On the contrary, miRNAs tend to be gene families, and thus previous approaches could generally overexpress only one member at a time (Sieber et al., 2007; Curaba et al., 2013).

Short tandem target mimic (STTM) can explore the functions of miRNAs by simultaneously blocking all the members in an miRNA family so as to achieve a gain-function of target genes through a single genetic transformation event (Yan et al., 2012). STTM is an artificial, short (~100 nt), non-coding RNA that is composed of two miRNA-binding sites and a spacer/linker. The two binding sites are complementary to the target small RNAs except for the central 3-nucleotide bulge, which sticks out between the 10th and 11th nucleotides of the targeted small RNA, so that the binding sites can trap miRNAs without being cleaved by them (Tang et al., 2012). STTM can be expressed through either stable plant transformation or virus-based transient expression systems (Sha et al., 2014; Teotia et al., 2017) and has been used to knockdown the target miRNA expression in *Arabidopsis* (Yan et al., 2012), soybean (Wong et al., 2014), tomato (Cao et al., 2016), cotton (Gu et al., 2014), and maize (Liu et al., 2019).

As one of the conserved miRNA families in plants, miR393 had been studied in several species like *Arabidopsis* (Navarro et al., 2006), rice (Bian et al., 2012), barley (Yuan et al., 2019), and poplar (Lu et al., 2008), and it was found that miR393 affected the plant root system (Vidal et al., 2010), leaf development (Si-Ammour et al., 2011), seedling growth and drought stress tolerance (Yuan et al., 2019), and salinity and alkaline stress (Gao et al., 2011). In *Arabidopsis*, miR393, which is encoded by MIR393a and MIR393b, can regulate its target genes including four F-box family auxin receptor genes (*Transport Inhibitor Response Protein 1, TIR1*) by splicing (Zhang et al., 2006; Si-Ammour et al., 2011; Wójcik and Gaj, 2016). *TIR1* family members affect different phenotypes including rooting, seedling growth, and seed coat development by regulating the auxin pathway (Parry et al., 2009; Hu et al., 2012). In poplar, the *FBL* family, which are homologous genes of the *TIR* family in *Arabidopsis*, have eight members, and *FBL1*-*FBL4* have been confirmed to be cleaved by miR393 (Shu et al., 2015; Tang et al., 2020). There are four members in the miR393 family of poplar, in which the mature miR393 encoded by different precursors share the same sequences (PmiREN, V2) (Guo et al., 2019). In a previous study, we analyzed the expression of miR393 in different tissues of poplar at multiple developmental stages and found that its expression was low in young tissues but high in mature tissues, especially in old stem (Tang et al., 2020). This suggests that miR393 may play a role in the vascular tissue development, which deserves further investigation.

In this study, we created miR393 STTM transgenic poplar (*Populus alba* × *Populus glandulosa*) in which the expression of miR393 was significantly reduced. The transgenic lines showed increased plant height, ground diameter, number of internodes, width of xylem, and total biomass production compared with the non-transgenic control plants. Accordingly, a large number of genes related to plant growth and wood formation were upregulated in STTM393 transgenic lines in order to inhibit the expression of miR393. The results support that blocking the function of miR393 by the STTM approach can promote plant growth and increase biomass production.

## Materials and Methods

### STTM393 Vector Construction and Transformation

The construction of the STTM393 vector was based on the previous method (Tang et al., 2012; Peng et al., 2018). Two primers with the sequences to form hairpin were synthesized according to the sequence of mature miR393, which is conserved across all the sequenced poplar species so far. The primer sequences were as follows: STTM393-Forward Primer: 5′-gcc**ATTTAAAT**atggtctaaagaagaaggatGATCAATGCGAtcgTCCCTTTGGAgaattcggtacgctgaaatcaccag-3′ and STTM393-Reverse Primer: 5′-gcc**ATTTAAAT**tagaccataacaacaacaacTCCAAAGGGAcgaTCGCATTGATCaagcttgggctgtcctctccaaatg-3′. The *SwaI* site “**ATTTAAAT**,” the *EcoRI* site “gaattc,” the *HindII* site “aagctt,” the artificial linker (20 nts) with the lowercase nucleotides, the “tcg” bulge in the middle of miR393 sequences, and the complementary sequences to d35S and T-35S of the pOT2 vector with lowercase nucleotides at the 3′-end were embedded in these two synthesized primers. Then, the pOT2 vector was used as a template for PCR amplification, and the linker PCR product was subjected to *SwaI* digestion and a subsequent self-ligation, which resulted in pOT2-STTM393. Finally, the pOT2 with STTM393 structure was sub-cloned into a binary vector pFGC5941, which was used to transform into 84K poplar (*P. alba* × *P*. *glandulosa*) by *Agrobacterium*-mediated leaf disk method. The positively transformed lines identified by resistance screening and expression analysis were used for further phenotypic observation.

### Plant Phenotypic Determination and Chemical Composition Measurement

The plantlets of STTM393 transgenic lines and 84K controls were grown in the culture medium for 1 month and then transferred to the soil pots. Seedlings were grown in a culture room under long-day conditions (16 h light/8 h dark) at 25/22°C (day/night) and in a greenhouse under natural light with daytime and nighttime temperatures of 24–30°C in the Chinese Academy of Forestry (Beijing, China). The plant height, ground diameter, and internode number were measured every month. The plant height is the length from the top of the plant to the base of the stem; the ground diameter is the diameter of the last internode at the base of the stem, and the number of internodes is the internode from the first expanded leaf to the last internode at the stem base. Stem tissues were sectioned by Leica VT1000S vibrating-blade microtome (Leica, Germany) with a thickness of 70 μm. The sections were stained by using toluidine blue (TBO) and phloroglucinol-HCl and were observed under Olympus BX51 microscope (Olympus, Tokyo, Japan). The stems of 6-month-old plants from 16th to 40th were used for cellulose, hemicellulose, and lignin content determination, according to the published protocol (Wang et al., 2020). Six STTM393 lines and 84K control plants were analyzed in this study, and each line included 6–10 vegetatively propagated individuals for the experiment. Data from each experiment were subjected to either an ANOVA or a *t*-test. All statistical analyses were performed using the SPSS Version 17.0 (SPSS, NY, United States). The phenotyping of STTM393 transgenic lines and 84K control plants was performed on three batches of plants.

### Total RNA Extraction and cDNA Synthesis

Total RNA was extracted using the LC Sciences Total RNA Purification Kit (#TRK-1001, LC Sciences, TX, United States) according to the previous modified methods (Tang et al., 2019). The integrity of total RNA was further assessed by 1.5% agarose gel electrophoresis, and the RNA concentration and purity were determined by NanoDrop™ 8000 Spectrophotometer (Thermo Fisher Scientific, MA, United States). Only RNA samples with an A260/A280 ratio between 1.9 and 2.1 and an A260/A230 ratio >1.80 were used for cDNA synthesis. We used a universal reverse transcription (RT) PCR method, in which the total RNA will be added by a poly(A) tail before RT (Tang et al., 2016). First, 2 μg of the total RNA was polyadenylated with ATP by poly(A) polymerase (PAP) at 37°C for 1 h in a 20-μl reaction mixture using the Poly(A) Tailing Kit (#AM1350, Invitrogen, MA, United States). Then, 10 μl (1 μg) of the E-PAP-treated total RNA was reverse transcribed with a poly(T) adapter universal RT-primer (5′-AAC GAG ACG ACG ACA GAC TTT TTT TTT TTT TTTV-3′) using PrimeScript™ RT reagent Kit (#RR037, TaKaRa, Shiga, Japan) following the instruction of the manufacturer. The cDNA was diluted 20-fold with nuclease-free water for quantitative real-time reverse transcription PCR (qRT-PCR).

### Quantitative Real-Time PCR Analysis

Quantitative real-time PCR was performed on the LightCycler^®^ 480 System (Roche Molecular Systems, Basel, Switzerland). The reaction mixture contained 10 μl of KAPA SYBR FAST qPCR Master Mix (# K4601, KAPA Biosystems, MA, United States), 2 μl of 20-fold diluted cDNA, 0.4 μM each of a forward and a reverse primer (Supplementary Table 1), and ddH_2_O in a final volume of 20 μl. Amplifications were performed with the following program: 95°C for 3 s; 40 cycles of 95°C for 10 s, 60°C for 30 s, and 72°C for 3 s. No-template reactions were used as negative controls, and *protein phosphatase 2A-2* (*PP2A-2*) and *polyubiquitin* (*UBQ*) were used as reference genes (Tang et al., 2019). Each sample was assessed in four technical replicates, and the experiment was repeated three times.

### High-Throughput Transcriptome Sequencing

The top five internodes of 3-month-old STTM393 lines (STTM393-2, STTM393-8, and STTM393-16) and 84K control plants were collected, among which 3–5 individuals in a line were randomly selected as a biological replicate, and each line had three biological replicates. The cDNA library construction and high-throughput sequencing of the above samples were performed by Biomarker Technologies (BMK, Beijing, China). The sequencing library used the NEBNext Ultra™ RNA Library Prep Kit for Illumina (NEB, MA, United States). The library was sequenced with Illumina Novaseq 6000, and the reading length was 2 × 150 bp (PE150). Clean data were obtained by removing the sequences containing adapter, poly-N, and low quality from raw data. Using Hisat2 software, the obtained clean data were compared with the genome of 717 poplar (*Populus tremula* × *P*. *alba*) (http://128.192.158.63/index.php/databases/spta-717-genome). The number of allowed mismatched nucleotides was set to “0” or “1.” After that, String-Tie (version 2.1.4) was used for transcriptional assembly and new transcriptional prediction, and the alternative splicing types and corresponding expression levels of each sample were obtained by ASprofile software. The quantification of gene expression level was estimated by the number of fragments per kilobase of transcript per million fragments (FPKM). EBSeq (Version 1.5.4) software was used to analyze the differential expression between STTM393 lines and 84K control samples. Genes with an adjusted FDR < 0.05 found by DESeq2 were assigned as differentially expressed. Gene ontology (GO) enrichment analysis of differentially expressed genes (DEGs) was carried out by GOseq R software, and the DEGs in the KEGG pathway were enriched by KOBAS software. The original transcriptome data were submitted to the NCBI public database Sequence Read Archive (SRA, PRJNA724789).

## Results

### STTM393 Promotes Poplar Growth

We obtained 30 STTM393 transgenic lines by *Agrobacterium*-mediated leaf disk method, and qRT-PCR showed that the expression of miR393 in 13 randomly selected lines was reduced over 10 times (Supplementary Figure 1). Six STTM393 transgenic lines with different expression levels were randomly selected for phenotype observation. The height, ground diameter, and internode number of these STTM393 transgenic plants at different developmental stages were significantly higher than those of 84K control plants (Supplementary Figure 2). Three STTM393 lines (i.e., STTM393-2, STTM393-8, and STTM393-16) with obvious phenotypic changes were used for further analysis.

**Figure 1:**
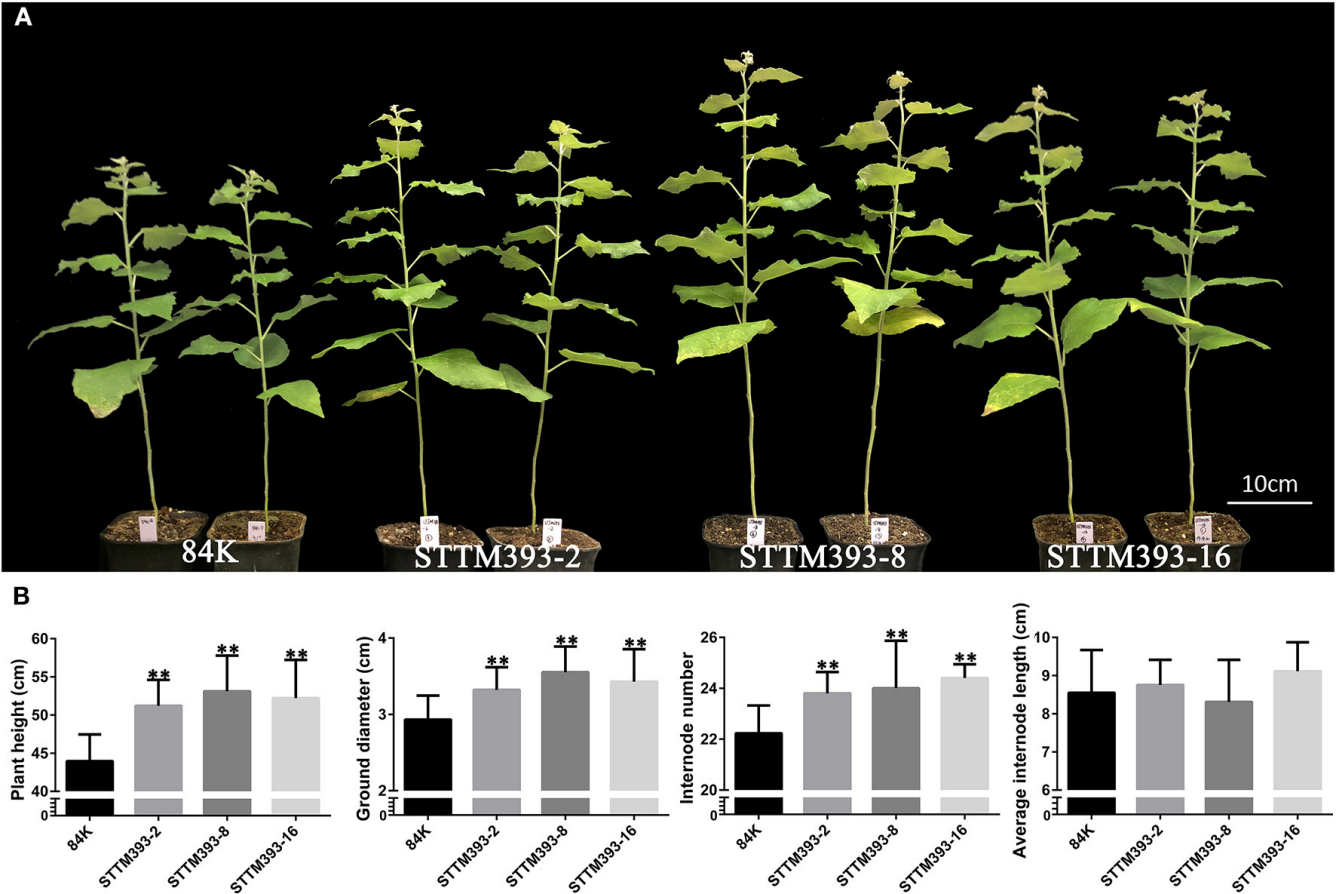
The phenotypes of STTM393 transgenic plants. **(A)** 3-month-old STTM393 and 84K control plants. **(B)** The height, ground diameter, internode number, and average internode length of 3-month-old STTM393-2, STTM393-8, STTM393-16, and 84K control plants. The bars represent means ± SD (*n* = 10), ^**^P ≤ 0.01.

The transgenic plants of STTM393-2, STTM393-8, and STTM393-16 grew faster than the control plants from tissue culture to soil cultivation. The plant height and root length of their 1-month-old tissue culture plantlets were longer than the control plantlets (Supplementary Figure 3A). Similarly, the STTM393 transgenic plants transferred to soil for 1 month (2-month-old plants) had significantly higher plant height, ground diameter, and internode number than those of the control plants (Supplementary Figures 3B–E). The height, ground diameter, and internode number of each 3-month-old STTM393 lines were 15–35, 15–25, and 7–13% higher than those of the control, respectively. However, the average internode length of STTM393 plants was not significantly different from that of the control plants (Figure 1). The three transgenic lines were grown three times, and similar results were obtained (Supplementary Figure 4). Therefore, the knockdown of miR393 family may promote plant growth in poplar.

### STTM393 Enhances Wood Formation

**Figure 2:**
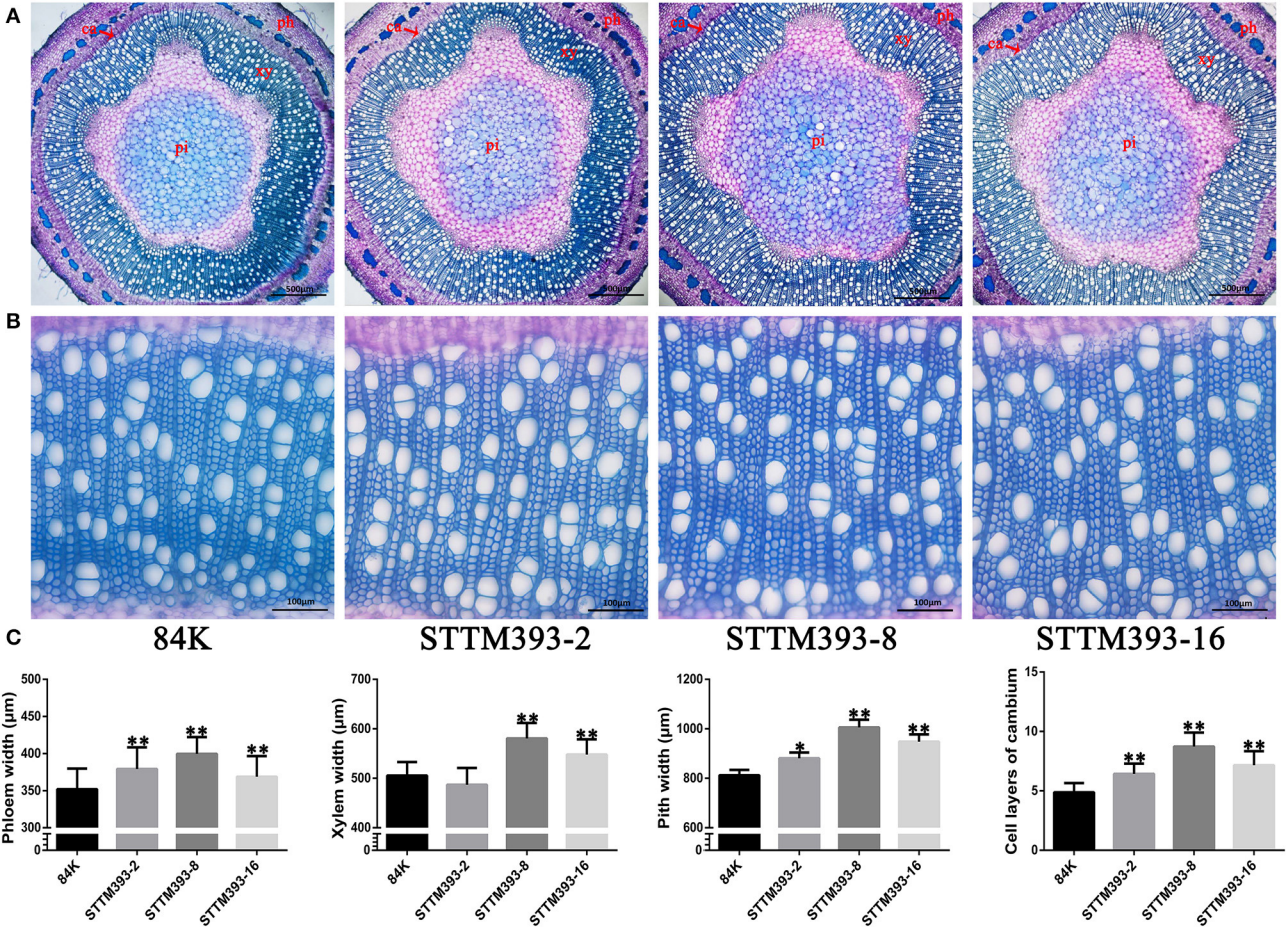
The vascular tissues in STTM393 transgenic plants. **(A)** The cross-sections of the 15th internode of 3-month-old STTM393 and 84K control plants stained by toluidine blue (TBO), Bar = 500 μm. ph, phloem; xy, xylem; pi, pith; ca, cambium. **(B)** The magnified xylem region of **(A)**, Bar = 100 μm. **(C)** The statistical data for phloem, xylem, pith, and cambium layers of 3-month-old STTM393 lines and 84K control plants. The bars represent means ± SD (*n* > 40). ^**^P ≤ 0.01, ^*^0.01 < *P* ≤ 0.05.

To investigate the function of miR393 in wood formation, we prepared the stem cross-sections to observe the vascular tissue of STTM393 lines. The results showed that the 15th internode of 3-month-old STTM393 transgenic plants was significantly larger in diameter than that of the control plants, while the cell layers of cambium and the width of phloem, xylem, and pith were also larger than of the control plants (Figure 2). This indicates that STTM393 transgenic plants can enhance the xylem development probably due to the high activity of cambium cells.

**Figure 3:**
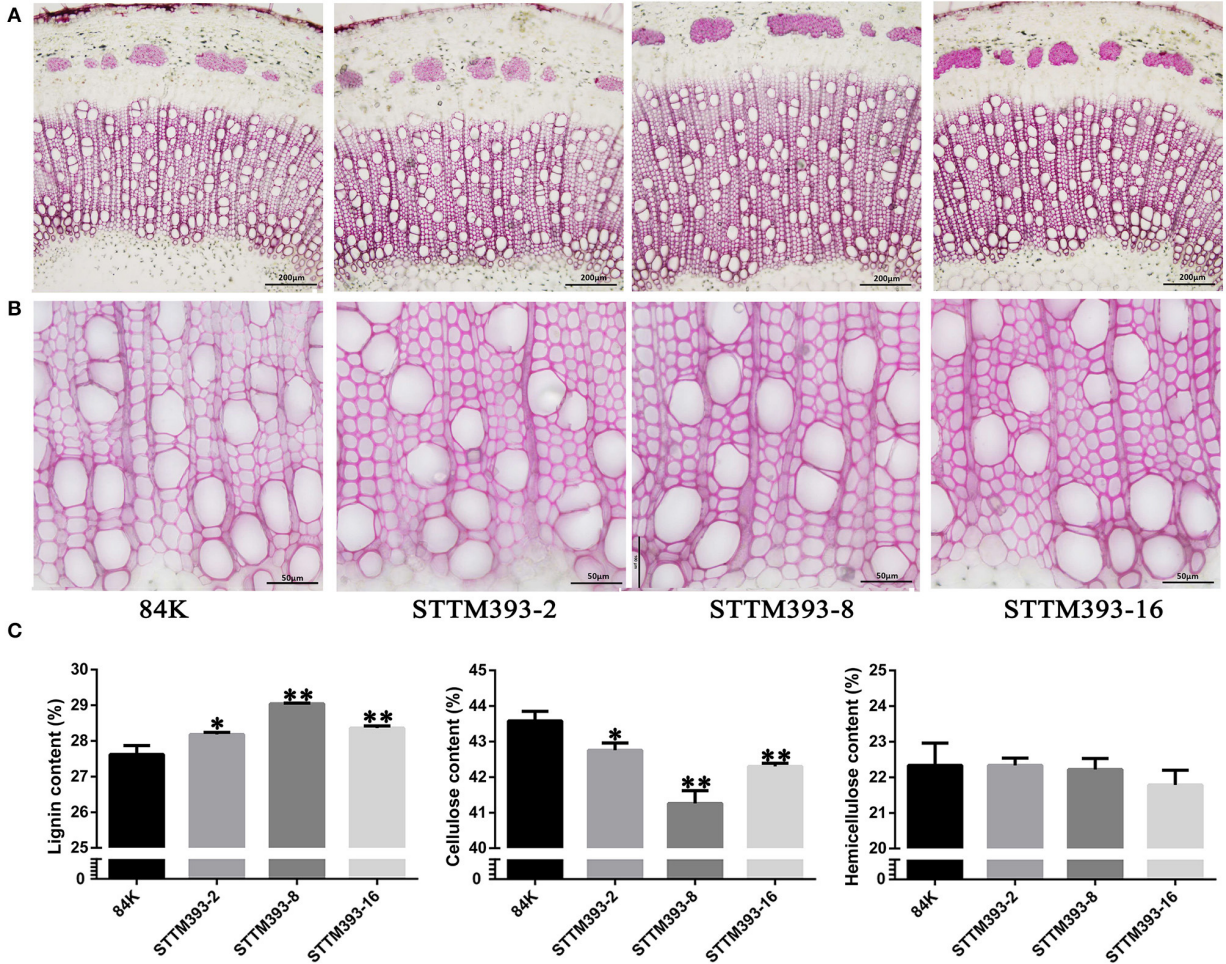
The chemical composition analysis in STTM393 transgenic plants. **(A)** The cross-sections of the 15th internode of 6-month-old STTM393 and 84K control plants stained by phloroglucinol-HCl, Bar = 200 μm. **(B)** The magnified xylem region of **(A)**, Bar = 50 μm. **(C)** The lignin, cellulose, and hemicellulose content of stem from 16th to 40th internodes of 6-month-old STTM393 lines and 84K control plants. The bars represent means ± SD (*n* > 6). ^**^P ≤ 0.01, ^*^0.01 < *P* ≤ 0.05.

Phloroglucinol staining showed that lignin deposition was increased in both the fiber and vessel cell walls of STTM393 transgenic lines, as revealed by the dark pink color (Figures 3A,B). Therefore, the content of lignin, cellulose, and hemicellulose in the stems of STTM393 transgenic lines and the controls was chemically determined. The STTM393 lines had significantly higher lignin content but lower cellulose content than the 84K controls (Figure 3C). However, there was no significant difference in the relative hemicelluloses content between STTM393 transgenic and control plants (Figure 3C). This suggests that the inhibition of miR393 expression may increase the lignin content in the secondary cell wall.

### STTM393 Increases the Expression of *PagFBLs*

**Figure 4:**
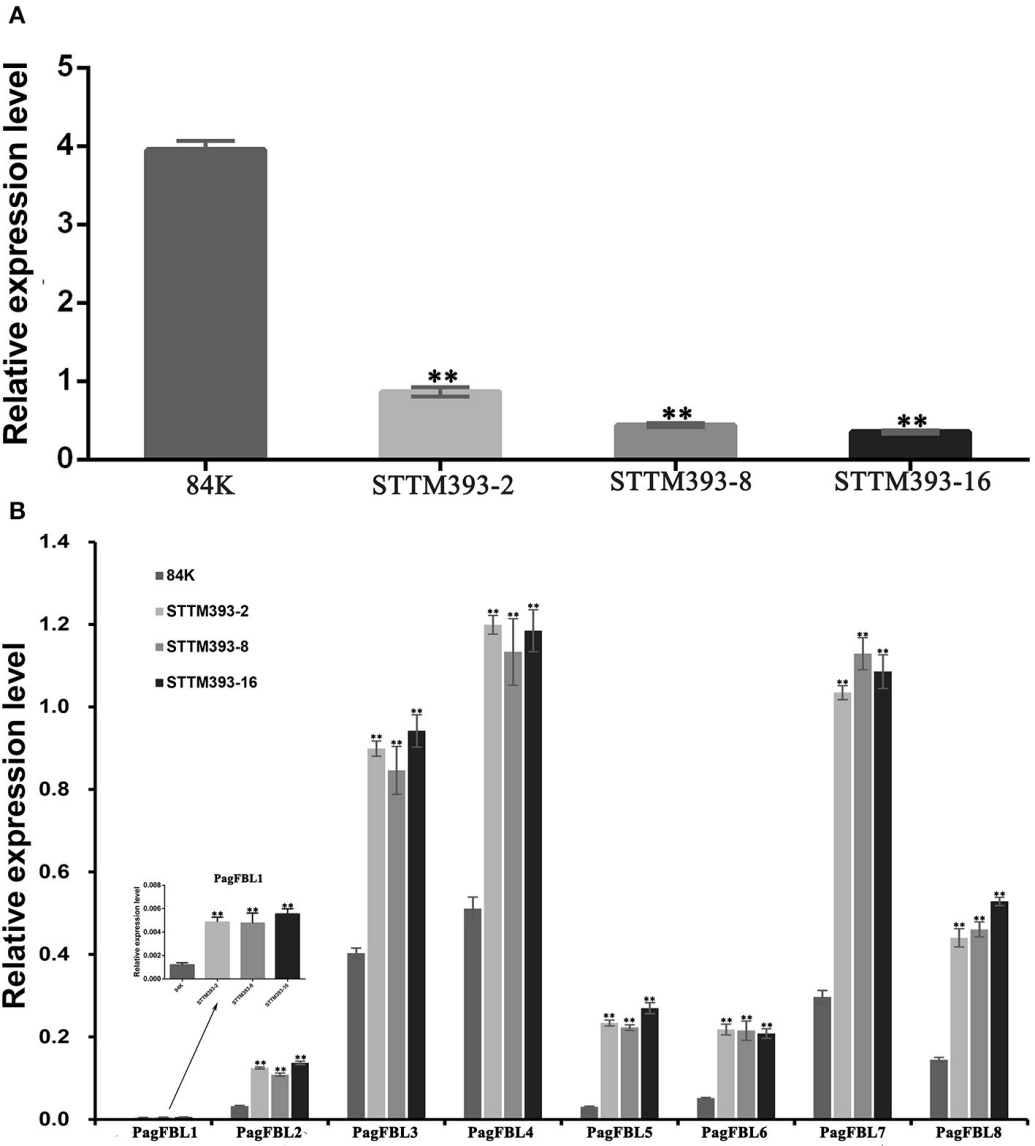
The expression of miR393 and *PagFBLs* in STTM393 lines by quantitative real-time reverse transcription (qRT)-PCR. The relative expression of miR393 **(A)** and eight members from *PagFBL* family **(B)** was detected by qRT-PCR in the 3-month-old STTM393 lines and 84K control plants. ^**^P ≤ 0.01.

We detected the expression levels of miR393 in the stems of 3-month-old STTM393 lines and 84K controls. The expression values of miR393 were reduced by 78, 88, and 91% in STTM393-2, STTM393-8, and STTM393-16 lines, respectively, compared with the control plants (Figure 4A). It indicates that the STTM of miR393 indeed causes the inhibition of the expression of miR393. *FBL* family members are the target genes of miR393 in poplar (Tang et al., 2020); thus, we detected *PagFBLs* expression in STTM393 lines and control plants by qRT-PCR and found that the expression levels of eight *PagFBL* genes were increased in STTM393 transgenic lines (Figure 4B). These results suggest that STTM393 can increase the expression of *PagFBLs* by inhibiting the function of miR393.

### Inhibition of miR393 Affects Genes Related to Plant Growth and Wood Formation

To understand which genes would be affected due to the inhibited miR393 expression, the expression of genes in three STTM393 lines (STTM393-2, STTM393-8, and STTM393-16) and control plants was detected by RNA sequencing. The results showed that there were 863 differentially expressed genes in the three STTM393 lines compared with the control, 431 of which were upregulated, and 432 were downregulated (Supplementary Table 2). The biological function of the upregulated genes was mainly enriched in the xyloglucan metabolic process (GO:0010411), cell wall biogenesis (GO:0042546), cell wall organization (GO:0071555), and response to auxin (GO:0009733), while that of the downregulated genes was mainly enriched in response to stimulus (GO:0050896), response to stress (GO:0006950), cellular response to sulfur starvation (GO:0010438), and response to an inorganic substance (GO:0010035).

Notably, the expression of genes related to auxin signal transduction had been upregulated, such as auxin-responsive protein (*SAUR1* and *SAUR78*), indole-3-acetic acid-amido synthetase (*GH3.6*), and indole-3-acetic acid-induced protein (*ARG7*). In addition, one auxin transporter protein, *AUX1*, and two homologous genes of the auxin-binding protein, *ABP19a*, had higher expression in STTM393 lines than in the control (Table 1). In STTM393 lines, several cell wall loosening factors including expansin (*EXP*), xyloglucan endotransglucosylase/hydrolase (*XTH*), glucan endo-1, 3-beta-glucosidase (*GLC*), and galacturonosyltransferase-like (*GATL*) were upregulated. Furthermore, the expression of cyclin genes regulating cell cycle transition was also upregulated, for example, cyclin D 1;1 (*CYCD1;1*), cyclin D 3;1 (*CYCD3;1*), and cyclin P 4;1 (*CYCP4;1*), all highly expressed in STTM393 transgenic lines. The higher expression of cell cycle-related genes may explain why the growth rate of STTM393 lines is faster than the controls.

**Table 1:** The differently expressed genes related to plant growth and wood formation.

**#ID**	**84K**	**S393-2**	**S393-8**	**S393-16**	**Change**	**Gene name**	**Annotation**
**Auxin signal transduction**							
Potri.008G066400.1	3.81	6.46	6.84	7.00	Up	AUX1	Auxin transporter-like protein 1
Potri.001G169000.1	90.36	339.12	255.85	213.42	Up	ABP19a	Auxin-binding protein
Potri.013G141900.1	507.95	1108.03	840.21	863.32	Up	ABP19a	Auxin-binding protein
Potri.004G165500.1	2.59	5.26	6.99	4.90	Up	SAUR1	Auxin-responsive protein
Potri.004G165600.1	0.74	2.11	4.28	4.30	Up	SAUR1	Auxin-responsive protein
Potri.003G071000.1	1.32	5.92	7.58	3.07	Up	SAUR78	Auxin-responsive protein
Potri.001G410400.1	4.10	7.14	8.73	8.19	Up	GH3.6	Indole-3-acetic acid-amido synthetase
Potri.011G129700.1	3.64	6.38	7.91	10.50	Up	GH3.6	Indole-3-acetic acid-amido synthetase
Potri.004G165800.1	0.60	2.26	2.69	4.33	Up	ARG7	Indole-3-acetic acid-induced protein
**Cell expansion**							
Potri.013G060800.1	21.93	41.26	46.23	44.81	Up	EXPA10	Expansin-A10
Potri.009G141400.1	2.63	11.30	10.54	9.52	Up	EXPL2	Expansin-like A2
Potri.002G236200.1	16.16	37.93	54.84	32.96	Up	XTH2	Xyloglucan endotransglucosylase/hydrolase 2
Potri.004G021000.1	18.89	60.70	65.11	43.95	Up	XTH8	Xyloglucan endotransglucosylase/hydrolase 8
Potri.019G125000.1	35.85	163.80	178.76	105.74	Up	XTH9	Xyloglucan endotransglucosylase/hydrolase 9
Potri.006G071200.1	7.73	40.09	23.95	21.20	Up	XTH23	Xyloglucan endotransglucosylase/hydrolase 23
Potri.018G095100.2	0.05	1.31	2.34	1.57	Up	XTH23	Xyloglucan endotransglucosylase/hydrolase 23
Potri.004G132700.1	1.22	2.01	2.15	2.70	Up	GLC11	Glucan endo-1,3-beta-glucosidase 11
Potri.008G055900.1	5.63	9.82	12.37	11.35	Up	GLC1	Glucan endo-1,3-beta-glucosidase 1
Potri.008G192600.1	0.79	2.20	3.02	2.26	Up	GATL9	Probable galacturonosyltransferase-like 9
Potri.010G038300.1	5.32	17.42	16.08	11.07	Up	GATL9	Probable galacturonosyltransferase-like 9
Potri.009G006500.1	21.21	43.10	45.58	28.49	Up	IRX7	Glucuronoxylan glucuronosyltransferase
**Cell cyclin**							
Potri.007G005700.1	0.72	1.71	2.75	1.63	Up	CYCD1;1	Cyclin-D1-1
Potri.009G086700.1	18.22	30.55	33.24	24.59	Up	CYCD1;1	Cyclin-D1-1
Potri.002G123000.1	8.96	12.58	16.04	13.59	Up	CYCD3;1	Cyclin-D3-1
Potri.012G115600.1	2.71	5.59	6.22	4.26	Up	CYCP4;1	Cyclin-P4-1
Potri.014G050400.1	21.52	56.45	69.92	44.15	Up	CYCP4;1	Cyclin-P4-1
**Lignin**							
Potri.002G004500.1	9.86	17.34	14.05	12.90	Up	CCR1	Cinnamoyl-CoA reductase 1
Potri.013G079500.2	2.64	9.01	9.23	6.20	Up	CCRL6	Cinnamoyl-CoA reductase-like
Potri.014G106500.2	1.70	3.07	2.72	3.48	Up	OMT1	Caffeic acid 3-O-methyltransferase
Potri.007G053400.1	9.25	18.01	17.71	12.44	Up	PER73	Peroxidase 73
Potri.002G031200.1	0.25	0.60	0.80	0.87	Up	PER9	Peroxidase 9
Potri.005G028200.1	12.56	34.36	38.49	25.00	Up	BEAT	Acetyl-CoA-benzylalcohol acetyltransferase
Potri.019G001400.1	2.99	6.26	7.54	5.16	Up	BEAT	Acetyl-CoA-benzylalcohol acetyltransferase
Potri.005G028500.1	9.96	27.35	29.87	20.98	Up	BEAT	Acetyl-CoA-benzylalcohol acetyltransferase
**Cellulose**							
Potri.005G087500.1	29.09	52.39	53.63	46.34	Up	CESA6	Cellulose synthase 6
Potri.018G029400.1	51.33	81.41	84.90	69.36	Up	CESA1	Cellulose synthase 1
Potri.003G142300.1	18.82	9.94	10.25	10.79	Down	CSLG3	Cellulose synthase like G3
Potri.006G004300.1	50.81	32.43	29.52	30.59	Down	CSLE1	Cellulose synthase like E1
Potri.006G004300.10	4.00	2.66	1.52	2.28	Down	CSLE1	Cellulose synthase like E1

In accordance with the relatively high content of lignin but low content of cellulose in STTM393 transgenic plants, the genes involved in the lignin synthesis pathway, such as cinnamoyl-CoA reductase (*CCR1* and *CCRL6*), caffeic acid 3-*O*-methyltransferase (*OMT1*), peroxidase (*PER9* and *PER73*), and benzylalcohol *O*-acetyltransferase (*BEAT*), were upregulated, while cellulose synthase-like genes (*CSLG3* and *CSLE1*) were downregulated (Table 1). The homologous genes of *CESA1* and *CESA6*, which are involved in the cellulose synthesis in the primary cell wall, exhibited higher expression in STTM393 lines. Therefore, the inhibition of the miR393 expression can increase the expression of genes related to lignification, thus promoting wood formation.

**Figure 5:**
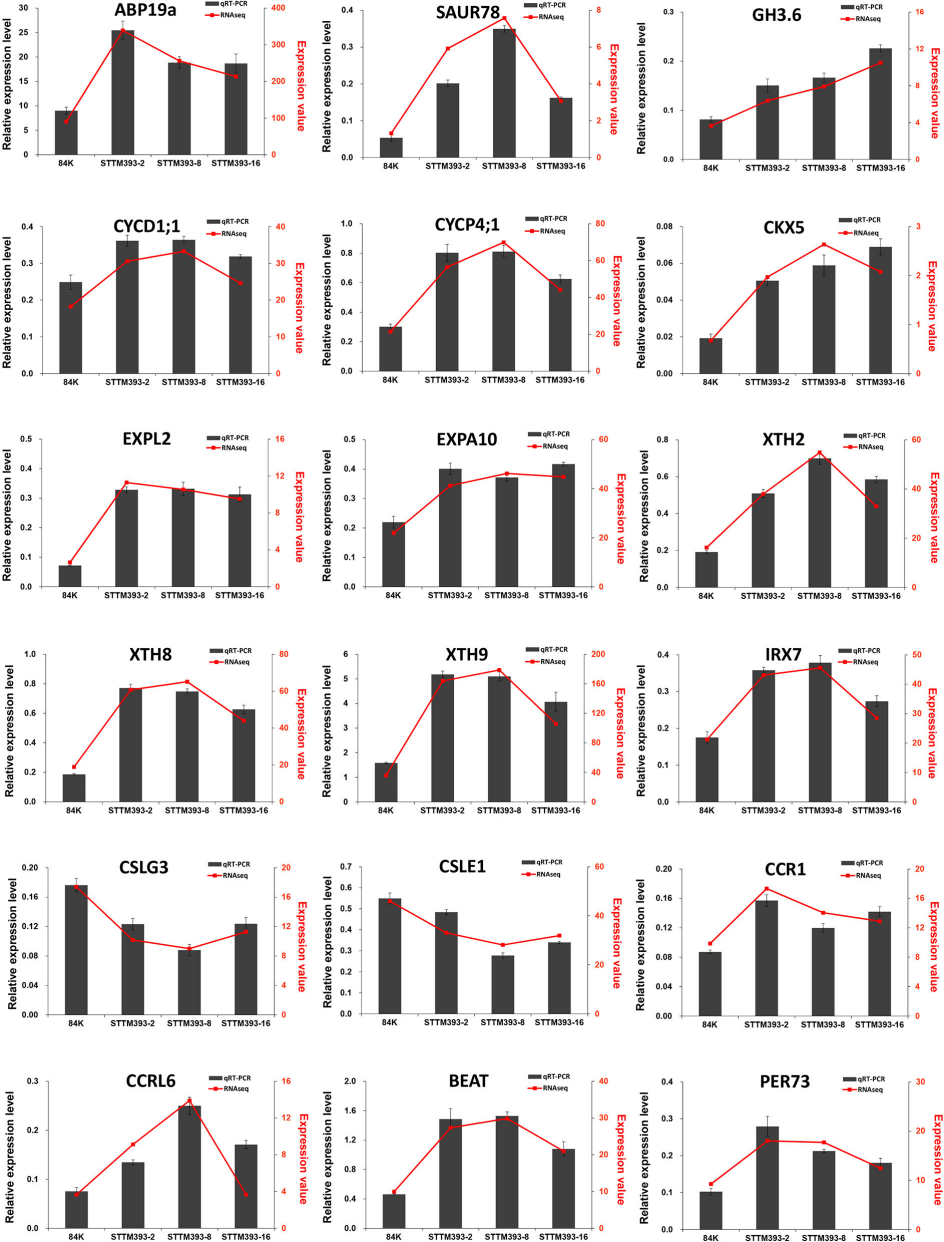
Validation of key genes related to plant growth and wood formation by qRT-PCR using *PP2A-2* as reference gene. The column represents the results of qRT-PCR, with the coordinate axis on the left (dark-grey). The line represents the results of transcriptome, with the coordinate axis on the right (red).

The expression levels of some genes mentioned above were verified by qRT-PCR. *PP2A-2* and *UBQ* were selected as reference genes for qRT-PCR. The expression trends of the genes calculated with *PP2A-2* (Figure 5) were consistent with those calculated with *UBQ* (Supplementary Figure 5). The relative expression levels of these genes obtained *via* qRT-PCR were consistent with that from the RNA sequencing data.

## Discussion

As an important regulator in the auxin signaling pathway, miR393 is involved in root development and stress resistance in herbaceous plants. For instance, the overexpression of miR393 can cause phenotypic changes in the length of main roots and the number of lateral roots in *Arabidopsis* and rice (Chen et al., 2011; Bian et al., 2012). We previously found that miR393 was highly expressed in developing secondary vascular tissues in poplar (Tang et al., 2016), but, so far, the role of miR393 in vascular tissue development has not been fully investigated. In this study, we blocked the miR393 expression in transgenic poplars using STTM technology and found its role in strengthening auxin signaling, which led to the promotion of secondary growth and production of high biomass in transgenic poplars.

To access the role of miR393 in secondary growth, we generated miR393 inhibition lines using the STTM approach. The plant height, ground diameter, and the number of internodes of STTM393 transgenic lines were all increased significantly, and this was opposite to the phenotype of the miR393 overexpression that inhibit apical meristem development observed in *Arabidopsis* (Wang et al., 2017). Interestingly, the cambium zone, pith, xylem, and phloem regions of the STTM393 lines were wider than the non-transgenic control plants, indicating that STTM393 could promote cell division, resulting in the increase of cell numbers in the pith, xylem, cambium, and phloem, which eventually led to the increase of stem diameter.

Previous studies had shown that miR393 in *Arabidopsis* and poplar played the role of splicing the target gene, *TIR1*, and its homologous, *FBLs* (Vidal et al., 2010; Chen et al., 2011; Tang et al., 2020). In this study, we found that the expression levels of all *FBL* family members were increased in the STTM393 lines, indicating that the function of miR393 on *FBLs* was successfully inhibited. A previous study suggested that *FBL1*-*FBL4* in *FBL* family members could be cleaved by miR393 (Tang et al., 2020). However, in this study, we found that the expression levels of all *FBL* members were increased using the STTM approach. These results suggest that miR393 may be tolerant to the mismatch with the target sequences. Overexpression of *PagFBL1* could promote the growth and root development in poplar (Shu et al., 2018); thus, the promoted secondary growth in STTM393 transgenic plants might be due to the increased expression of *FBL* family members.

Auxin is a very important hormone in controlling plant growth and development, and the FBL family members as putative auxin receptors mediate the auxin signaling pathway. To further investigate the role of auxin signaling in the development of secondary vascular tissues, we used RNA sequencing to check the genes changed in expression due to the inhibition of miR393. The downstream genes of the auxin signaling pathway, such as *SAUR78* and *GH3.6*, were upregulated, of which *GH3.6* was known to be regulated by *PagFBL1* and might modulate adventitious rooting (Shu et al., 2018), while the overexpression of *SAUR78* promoted plant growth in *Arabidopsis* (Li et al., 2015). Several cyclin genes, including *CYCD* and *CYCP*, were also upregulated in the STTM393 lines. Cyclin D can regulate cell transformation from the G1 phase to the S phase to start the cell cycle by promoting DNA synthesis and cell proliferation (Lu et al., 2011). Overexpression of *CYCD2* in tobacco could increase the rate of plant growth and biological yield by accelerating the rate of cell division (Cockcroft et al., 2000). Similarly, *CYCD3* was a positive regulator of cambial cell proliferation and secondary growth (Collins et al., 2015). Therefore, the strengthened auxin signaling also leads to the activation of cell cycles to accelerate cell division and thus promote the secondary growth of poplar.

Plant cell wall loosening is a necessary physiological process during cell expansion and elongation throughout the entire growth and development of plants. It is due to the hydrolysis of polysaccharides in the cell wall caused by the action of cell wall loosening factors (Zhao and Li, 2011). The expression levels of several cell wall loosening factors, such as *EXPA10, XTH2, XTH8, XTH9*, and *XTH23* genes, were upregulated in the transgenic lines. Previous studies have shown that *EXPA* is involved in the regulation of xylem formation (Gray-Mitsumune et al., 2004), while *XTH* family members are involved in the cell wall synthesis in poplar (Mellerowicz and Sundberg, 2008), but *XTH9* mutation leads to shorter internode length (Hyodo et al., 2003). The upregulation of these genes provides support to the observed fast xylem development in transgenic lines. In addition, the lignin synthesis pathway genes, *CCR1, CCRL6, PER73*, and *BEAT*, were upregulated in the STTM393 lines, in comparison with the control plants. The increased expression of key enzymes in the lignin synthesis pathway can promote lignin synthesis (Xie et al., 2018). On the contrary, the expression levels of two cellulose synthase-related genes, *CSLG3* and *CSLE1*, in STTM393 lines were decreased. This was in accordance with the observation that STTM393 lines exhibited higher lignin content but lower cellulose content in stems.

In summary, the STTM approach can block the function of miR393, thus increasing the expression of its target genes *FBLs*. The strengthened auxin signaling promotes secondary growth of and biomass production in transgenic poplars, by changing a series of downstream genes orchestrating cambium activity, xylem development, and cell wall synthesis. This study provides additional support, that is, manipulating auxin signaling regulators could enhance trees for fast growth and biomass production.

## Data Availability Statement

The datasets presented in this study can be found in online repositories. The names of the repository/repositories and accession number(s) can be found below: SRA, PRJNA724789.

## Author Contributions

FT designed the study. FT constructed the STTM393 vector and LC transformed it into poplar. FT, XH, and LC investigated the growth data of STTM393 transgenic and control plants. FT and XH completed the section and microscopic observation of different stem nodes of STTM393 plants. FT and LC analyzed the data and drafted the manuscript. WS and LW helped in the interpretation of results. All of the authors carefully checked and approved this manuscript.

## Conflict of Interest

The authors declare that the research was conducted in the absence of any commercial or financial relationships that could be construed as a potential conflict of interest.
