# Supplementary material for: Knockdown of miR393 Promotes the Growth and Biomass Production in Poplar
Source: Front Plant Sci. 2021 Jul 14;12:714907. doi: 10.3389/fpls.2021.714907 (PMC8317616; doi:10.3389/fpls.2021.714907)
Supplement: Supplementary file 1 [file Data_Sheet_1.PDF]

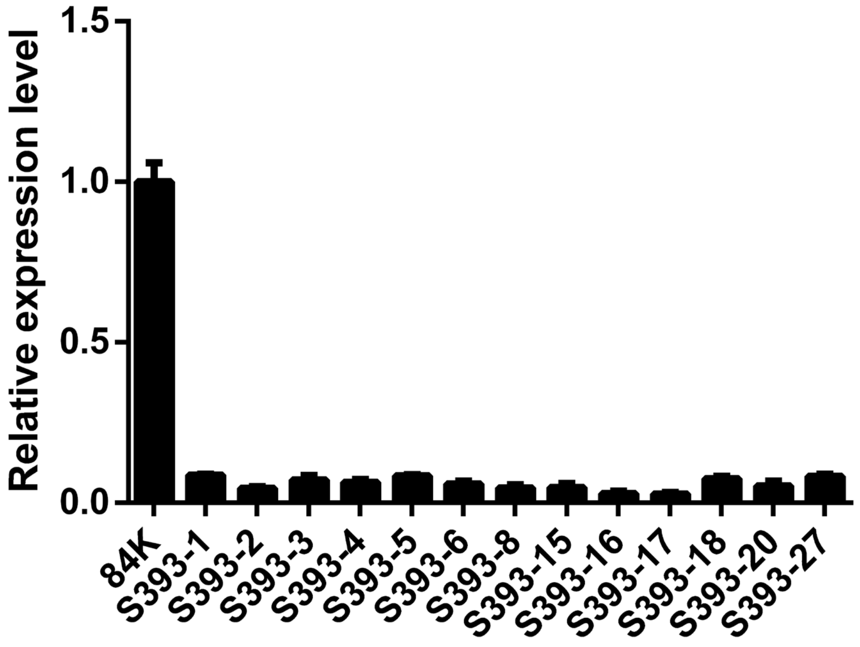

Supplemental Figure S1. The relative expression of miR393 in STTM393 transgenic lines detected by qRT-PCR

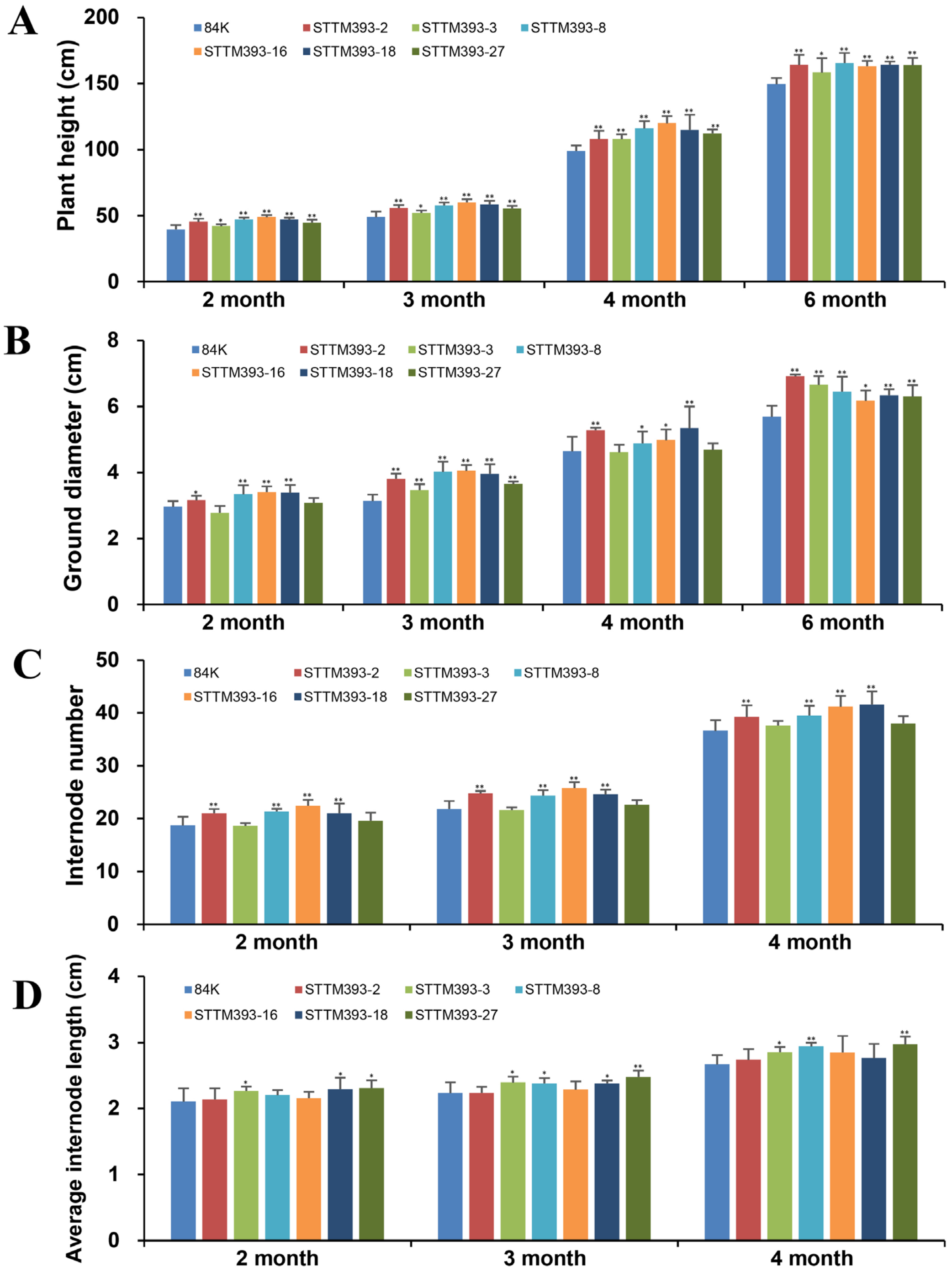

Supplemental figure S2. The growth of STTM393 transgenic plants
(A) The plant height, (B) ground diameter, (C) internode number and (D) average internode of STTM393 lines and 84K control plants at several developmental stage. The bars represent means ± SD (n=6).** $P \leq 0.01$; * $0.01 < P \leq 0.05$.

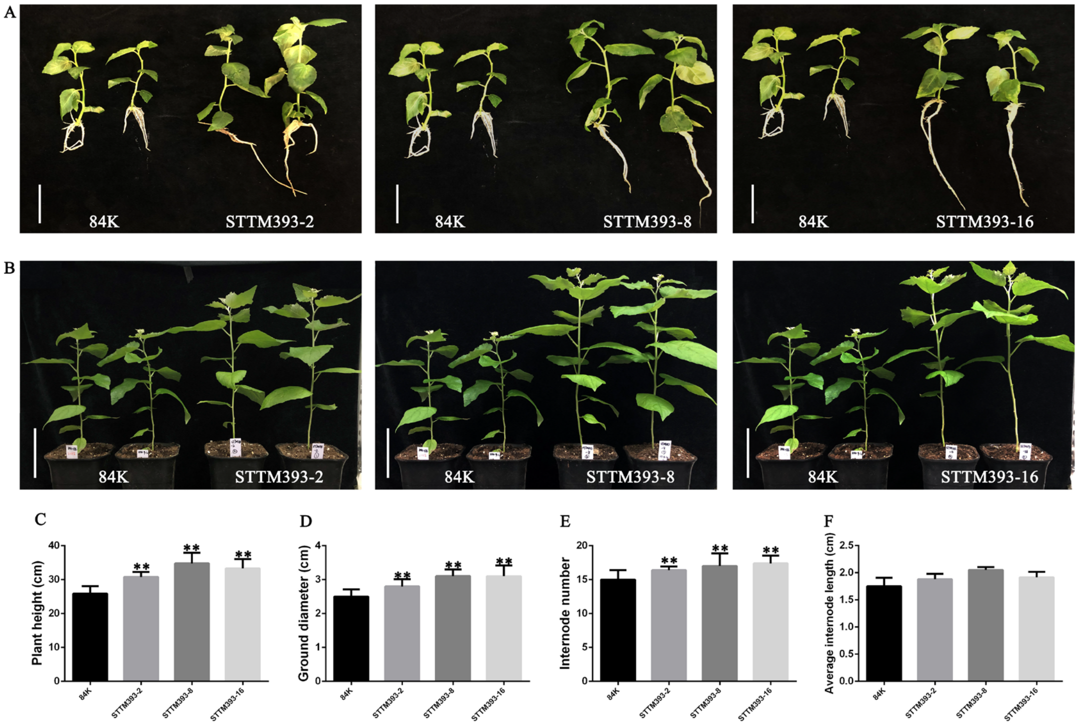

Supplemental figure S3. The phenotype of STTM393 transgenic plants at different developmental stages

(A) The phenotype of 1-month seedlings in tissue culture, Bar=3cm; (B) The phenotype of 2-month plants in soil culture, Bar=10cm. (C-F) The height, ground diameter, internode number, and average internode length of 2-month STTM393 transgenic lines and 84K control plants. The bars represent means ± SD (n=6). ** $P \leq 0.01$.

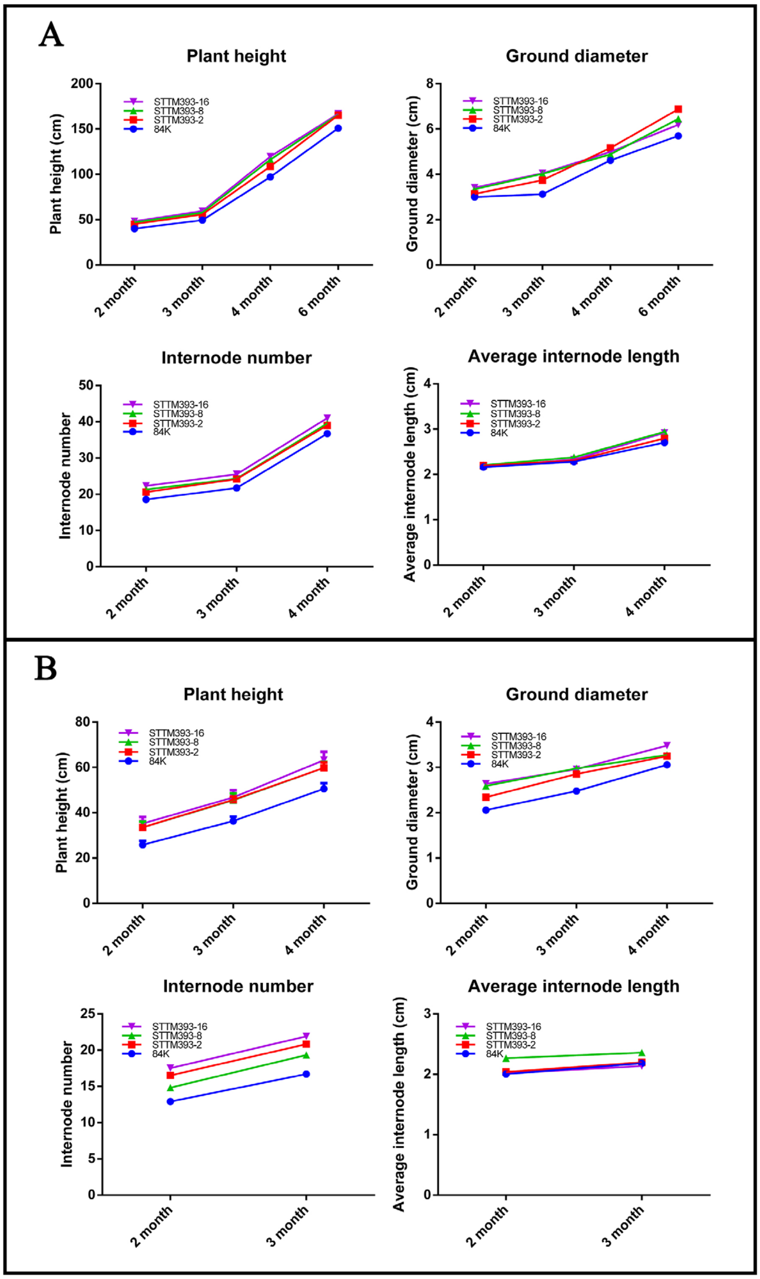

Supplemental figure S4. The growth of STTM393 transgenic plants from different batches

The plant height, ground diameter, internode number and average internode length of STTM393 lines and 84K control plants at several developmental stages from (A) first batch and (B) second batch.

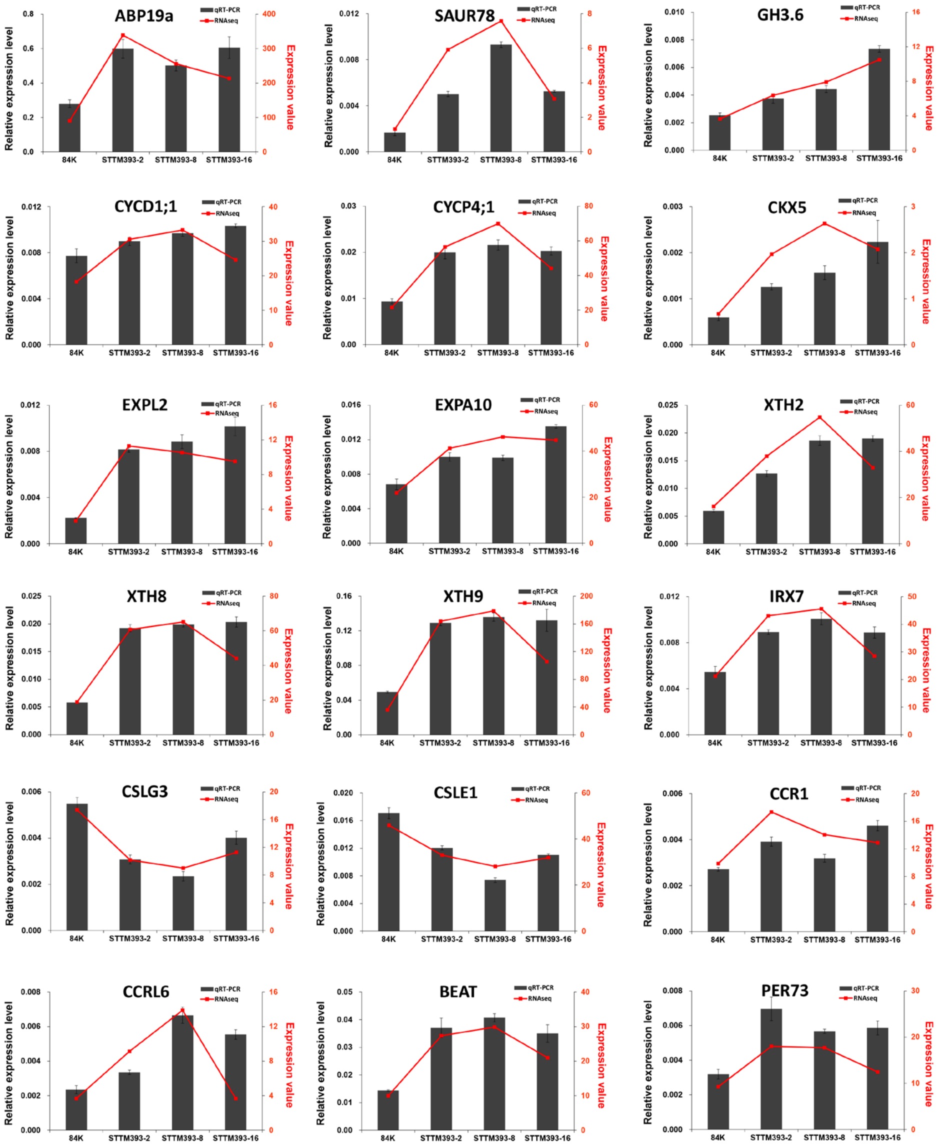

Supplemental figure S5. Validation of key genes related to plant growth and wood formation by qRT-PCR using *UBQ* as reference gene

The column represents the results of quantitative PCR, with the coordinate axis on the left (black). The line represents the results of transcriptome, with the coordinate axis on the right (red).
